# Predicting Ancestral Segmentation Phenotypes from Drosophila to Anopheles Using *In Silico* Evolution

**DOI:** 10.1371/journal.pgen.1006052

**Published:** 2016-05-26

**Authors:** Jeremy B. Rothschild, Panagiotis Tsimiklis, Eric D. Siggia, Paul François

**Affiliations:** 1 Physics Department, McGill University, Ernest Rutherford Physics Building, Montreal, Quebec, Canada; 2 Center for Studies in Physics and Biology, The Rockefeller University, New York, New York, United States of America; New York University, UNITED STATES

## Abstract

Molecular evolution is an established technique for inferring gene homology but regulatory DNA turns over so rapidly that inference of ancestral networks is often impossible. *In silico* evolution is used to compute the most parsimonious path in regulatory space for anterior-posterior patterning linking two Dipterian species. The expression pattern of gap genes has evolved between *Drosophila* (fly) and *Anopheles* (mosquito), yet one of their targets, *eve*, has remained invariant. Our model predicts that stripe 5 in fly disappears and a new posterior stripe is created in mosquito, thus *eve* stripe modules 3+7 and 4+6 in fly are homologous to 3+6 and 4+5 in mosquito. We can place *Clogmia* on this evolutionary pathway and it shares the mosquito homologies. To account for the evolution of the other pair-rule genes in the posterior we have to assume that the ancestral Dipterian utilized a dynamic method to phase those genes in relation to *eve*.

## Author Summary

The last common ancestor of the fruit fly (*Drosophila*) and mosquito (*Anopheles*) lived more than 200 Million years ago. Can we use available data on insects alive today to infer what their ancestor looked like? In this manuscript, we focus on early embryonic development, when stripes of genetic expression appear and define the location of insect segments (“segmentation”). We use an evolutionary algorithm to reconstruct and predict dynamics of genes controlling stripes in the last common ancestor of fly and mosquito. We predict a new and different combinatorial logic of stripe formation in mosquito compared to fly, which is fully consistent with development of intermediate species such as moth-fly (*Clogmia*). Our simulations further suggest that the dynamics of gene expression in this last common ancestor were similar to other insects, such as wasps (*Nasonia*). Our method illustrates how computational methods inspired by machine learning and non-linear physics can be used to infer gene dynamics in species that disappeared millions of years ago.

## Introduction

Molecular phylogenies based on protein coding genes have greatly enhanced evolutionary theory, and in favorable cases even allow a reconstruction of the last common ancestral gene or even full evolutionary pathways [1]. However regulatory sequence evolves more rapidly than coding sequence and the functional binding sites can move around without impacting the function of a ∼1kb functional regulatory module [2, 3]. Thus one is often in the situation where gene homologies are obvious, yet there is no visible sequence homology in the regulatory regions. At the phenotypic level, gene expression domains can be easily mapped by in-situ hybridization yet a molecular understanding is limited outside of model organisms. There is considerable need for a computational tool that can take sparse phenotypic information, e.g., broadly defined space-time gene expression, and construct the simplest phylogenetic relationships consistent with data, thereby highlighting interesting events for molecular follow up.

*Drosophila* segmentation is a paradigmatic example of dynamic developmental network. Positional information propagates from maternal gradients such as *bicoid* (*bcd*) and *caudal* (*cad*) to gap genes such as *hunchback*, *giant*, *knirps* and *Kruppel* (respectively *hb*, *gt*, *kni*, *Kr*), and then to the striped expression of primary pair-rule genes such as *even-skipped* (*eve*), *hairy* (*h*), *runt* (*run*), and partially *fushi-tarazu* (*ftz*) [4, 5]. The pair-rule genes in turn control the segment polarity genes that are broadly conserved across the arthropods [6]. Mutagenesis and bioinformatics studies have revealed the main DNA motifs controlling the expression of gap and pair-rule genes [7] while systematic quantitative imaging has led to phenomenological models for segmentation dynamics [8, 9].

Recent evo-devo studies have started to map the segmentation hierarchy in other dipterans (*Anopheles* [10], *Clogmia* [11], *Megaselia* [12]). Almost all information comes from localizing the relevant mRNA by in-situ hybridization, and knocking down (KD) various transcripts with RNA interference. Information in each of these three species is still very sparse: while we know the position of the gap genes and the single pair-rule gene *eve*, there is only few information on the phasing of the other pair-rule genes relative to *eve*. Whether they are positioned by the gap genes or other so-called primary pair-rule genes is not known in those Dipterans. There are no defined gene regulatory modules in these species, so all information about gap gene regulation is inferred from their position and shifts in putative targets under KD.

In spite of this sparse information, some interesting questions can be posed. The anterior gap gene pattern appears invariant in all species as do the *eve* stripes, though there are only six in *Clogmia* before gastrulation vs 7 in *Drosophila* and up to 8 in *Anopheles*. There is more variability in the posterior. The relative positions of the posterior domains of *hb* and *gt* are inverted in *Anopheles* with respect to *Drosophila*, while in *Clogmia*, neither of these gap genes are expressed posteriorly before gastrulation. It is reasonable to assume that the primary pair-rule stripes are positioned by gap gene repression, so the evolutionary interchange of the posterior *hb* and *giant* domains poses problems for individual *eve* stripe regulatory modules. For instance, *eve* 5 in *Drosophila* is repressed posteriorly by *gt* so if the posterior *gt* domain is removed, *eve* 5 extends broadly posteriorly in *Drosophila* [13]. So how can *gt* domain be much more posterior in *Anopheles*, and virtually nonexistent in *Clogmia*? Similarly the two nested modules *eve*3+7 and 4+6 are both defined by *kni* repression from the interior and *hb* repression from the exterior [14], which seems less plausible in *Anopheles* based on the relative positions of the *eve* stripes and gap genes.

How is computational modeling best harnessed to the task of inferring the evolutionary path between fly and mosquito with such sparse information about one endpoint and intermediates? One very general lesson from the machine learning field is to avoid overfitting [15] [16]. More parameters make less predictive, “hairball” models [17] that can always be complexified rather than falsified. The temptation in the present instance is to import into the evolutionary simulation all the molecular details we have accumulated about *Drosophila*. A realistic model for the AP patterning in *Drosophila* with multiple factors, short range repression and cooperativity, was formulated in [18], and applied to the evolution of new enhancers in [3]. When guided by *strong selection* for the correct domain of expression [3], new modules can evolve on the time scale of 10^7^ years [19]. The key point made in these and related papers is that de novo evolution of enhancers is fast because their genotype to phenotype map can be optimized by point mutations and hill climbing. These papers also observe that under the quick and sloppy logic of evolution, the excess of binding sites or the prevalence of generic activators and position specific inhibitors can all be understood as the most quickly realized solutions to the fitness optimization problem.

We do not see the creation of new modules in response to strong selection as necessary for the transition from fly and mosquito back to their last common ancestor (LCA). Rather via the logic of evolutionary *bricolage* [20], organic evolution and thus computation, should seek the most quickly evolved repurposing of existing components that connects the two defined endpoints subject to the constraint of viability for all intermediates. We will show that gap and pair-rule regulation in fly can be continuously adjusted to accommodate the observed changes in the posterior gap gene expression patterns. Given the range of times we have to cover, the high rate of churn in regulatory sequence among the Drosophlids [19](with little effect on phenotype), and the changes in regulatory factors such as the absence of *bicoid* in *Anopheles*, it is thus most practical and informative to simulate the phenotype and ignore the molecular level.

Phenotypic models have been informative in other areas [21, 22] and in the present context fit quantitative genetic data as to how expression domains shift when upstream factors are altered. Similar approaches are found in [23], and [8]. We then use an evolutionary computation that initializes the network model with *Drosophila* parameters, and mutates and selects with a ‘fitness’ that directs the model towards *Anopheles*. Putting aside the specific molecular information we have for *Drosophila* makes our approach applicable to a wider range of problems.

Invariably we find, *eve* stripe 5 disappears and either (or both) the *eve* 4+6 or 3+7 modules add a third posterior stripe to compensate. Thus the posterior *eve* stripes are not homologous in *Drosophila* and *Anopheles*. When we consider regulation of the other primary pair rule genes in fly, we conclude that the most plausible common long-germ ancestor of fly and mosquito employed a dynamic patterning system based on a forward shift in the *eve* pattern as observed in *Clogmia* and *Drosophila* [24] to impose phase relationships on the remaining pair rule genes. Thus there should be no homology in the posterior gap gene regulation of *run*, *h*, or *ftz* between fly and mosquito.

We emphasize that no computation, no matter how complex, will ever *prove* one evolutionary scenario over another. Computation is at best a heuristic tool to uncover interesting hypothesis that one could not guess, and buttress those hypothesis by their fidelity with a quantitative phenotypic model for regulation. The computation is like a screen for all solutions to an evolutionary problem given defined rules. To the extent the ingredients of the phenotypic model are plausible and transparent, and the predictions intuitive, they may stimulate experiments.

## Materials and Methods

### Evolutionary algorithm

The main lesson of two decades of quantitative analysis of *Drosophila* segmentation is that positional information of pair-rule stripes is essentially defined by gap-gene repression (see e.g. review in [5]). Gap genes themselves are positioned by a mixture of cross-repression [9, 25] and activation provided by maternal gradients. We will build our genetic or phenotypic model for *Drosophila* by defining an interaction kernel for each gap gene and pair-rule regulatory module. The kernel takes the numerical values of the inputs and outputs the expression. The general functional form is given in the S1 Text, and the specific inputs shown in the next subsection.

The evolutionary algorithm that will produce the *Anopheles* network is allowed to change only the numerical parameters within the kernel functions. Thus the parameters that define the maternal to gap regulation, interactions among the gap genes, and their regulation of the pair rule genes all change. The algorithm does not create new kernels nor add new inputs to existing kernels, but it is important to include from the start all potential regulatory inputs that might play a role during evolution, even if their effect is minor in *Drosophila*. The output of a kernel is allowed to become 0 signifying its elimination.

This conservative choice for the allowed ‘mutations’, was motivated above, and justified here. Firstly we show that the desired conversion from fly to mosquito can be realized without adding new kernels, and merely modifying existing ones. Binding sites turn over rapidly in modules so parameter evolution in existing kernels should be fast, while creating kernels in the absence of directional selection in anticipation of a future need is more speculative, and arguably slower. Secondly the anterior (roughly *eve* stripe 4 and forward) gap gene pattern in *Anopheles* and the intermediate species is largely invariant, while several of the pair-rule gene modules control both an anterior and a posterior stripe. Since we will impose that the anterior regulation is invariant, it was most logical to keep the inputs to these two stripe kernels invariant also. Once the allowed mutations are defined, the algorithm proceeds by rounds of mutation-selection. A population of networks is initialized to the *Drosophila* parameters, each network is mutated and retained if it is more fit than its parent. The most fit half of the population is duplicated and forms the next generation. Details on the code can be found in [26, 27], and our code is available upon request.

The function (negative fitness) that we want to minimize for each network is a sum of terms measuring (1) deviation of the posterior *hb* and *gt* profiles from the *Anopheles* pattern (2) deviation of the anterior *eve* profile from *Drosophila*. In addition there must be at least 7 *eve* stripes. From [10] we know *hb* moves forward in mosquito, while posterior *gt* is weak and probably plays no role in patterning so we assume it’s absent. Note we constrain the expression profiles, so evolution has to find a way to alter the posterior *hb* kernel to move its expression forward and match *Anopheles*. When we include a second pair rule gene, *ftz*, to define the 14 stripe segment polarity pattern, we insist its stripes alternate with those of *eve*. Nothing about intermediate species such as *Clogmia* is assumed. Once the evolutionary path to *Anopheles* is understood, and with it the regulation, the homology of the 6 *Clogmia* stripes becomes obvious without any further computation as we explain below.

We do not impose that the eve stripes be of equal width, though in a number of instances we checked that local parameter optimization can readily satisfy this constraint, see e.g. S4 Video. Normal *Drosophila* segmentation is known to be extremely precise, [28, 29]. However considerable change in *eve* expression in the blastula is not incompatible with adult viability. An early example was induced by variable *bcd* dosage [30]. Later examples include loss of parasegments 7 and 11 [31], and even abdominal segment A5 [32, 33], with further details left for the discussion. Some variation in phenotype is essential for evolution. Since one can only claim heuristic value for our evolutionary computations, trying to better define the fitness costs of quantitatively imperfect patterns adds more uncertainty than it resolves and encumbers a simple story.

### Idealized *Drosophila* network

**Fig 1:**
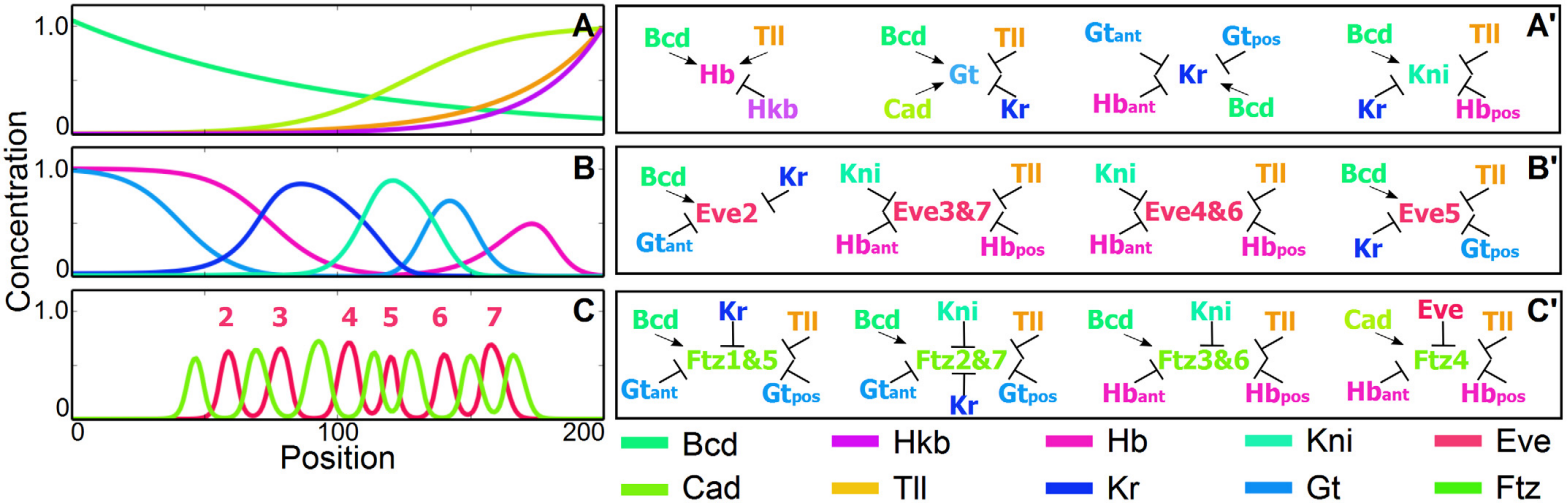
Simplified model of *Drosophila* network. A-B: simulated maternal and gap gene profiles. C: simulated pair-rule gene profiles. A’-C’ Summary of interactions used to generate these profiles. Equations and references for the interactions are given in the S1 Text. A generic spatially uniform activator is assumed where needed.

The starting point of our simulations is an idealized *Drosophila* shown in Fig 1. S1 Text details our assumptions, we summarize their main features below.

There are maternal input gradients, *bcd* anterior and *cad* posterior, repressed by *bcd*. *bcd* is frozen throughout the simulation. While there is no *bcd* in mosquito, we assume some other gene such as *otd* takes its place [34]. In addition we have fixed profiles of *tailless* (*tll*) and *huckebein* (*hkb*) in the posterior. Those gradients supply positional information to the gap genes, *hb, gt, kni, Kr*, which are the only ones we need to follow during the evolution. At the phenotypic level we consider, gap domains look very similar in *Drosophila* and *Anopheles*, the main difference being the posterior exchanges between *hb* and *gt*.

Our description of the kernels defining gap territories incorporates regulatory interactions inferred from genetics, that are presumably conserved in evolution given the observed similarities of the gap patterns (see details in S1 Text). Repression comes from more than the immediately adjacent gap genes, since when these are mutated, expression typically does not extend to the anterior or posterior pole of the embryo. We omit other potential interactions because they do not impact the conserved qualitative gap pattern and would further require detailed molecular data to be fit in a species-specific manner [9, 35]. The reader will observe that all gap gene expression patterns along the computed pathways from fly to mosquito remain fixed in size, suggesting we are not omitting any essential interactions as *gt* and *hb* interchange.

For *eve* we include only stripes 2 to 7. (We do not simulate *eve* stripe 1 because we focus on the posterior regulation, and its regulation is decoupled from the other stripes.) and thus have four *eve* modules to consider *eve* 2 [36], *eve* 3+7, *eve* 4+6 [14, 37] and *eve* 5 [13]. There is good genetic evidence, reinforced by bioinformatic studies [5, 7], that their position is largely defined by gap gene repression. We include more than the minimal interactions required to fit the wild type *eve* and gap gene patterns in the posterior since *hb* and *gt* domains interchange as we evolve to mosquito, and mutagenesis experiments in fly suggest stripe regulation by more than the closest gap genes. For instance, in a *hb* mutant background, neither *eve* 6 nor *eve* stripe 7 expand much in the posterior [13] and in a *gt* mutant, *eve* 5 stripe only extends posterior to *eve* 7 stripe [13]. Thus there must be additional repression from the posterior that we assume comes from *tll*. We allow a uniform activator for stripes 3–7 and 4–6 (supplied by DSTAT [7, 37] or Zelda [38, 39]), but in our framework no positional information is given by activators.

There are a similar set of *ftz* modules defined by gap gene repression in Fig 1. *ftz* 4 represents a special case in that there is no stripe specific element and it appears that *ftz* stripe 4 is only expressed as part of the 7 stripe ‘zebra’ element [5]. Thus *ftz* has partially the character of a secondary pair rule gene that takes input from other primary genes, a fact that will be important in the following.

## Results

Gap gene *Anopheles* pattern has been described by Goltsev et al. [10]. As explained before, the main difference between *Drosophila* and *Anopheles* gap patterns is in the relative positioning of *hb* and *gt*. Specifically *hb* moves forward and the *gt* domain becomes so posterior in *Anopheles* that it is unlikely to set stripe boundaries.

This interchange of localization in the course of evolution poses a problem for *eve* 5 whose posterior boundary is regulated by *gt* in *Drosophila* which is implausible in *Anopheles*, and also in *Clogmia* where posterior *gt* is absent (and therefore in the LCA of these three insects). So it is very plausible that *eve* regulation has changed between these insects. Similarly, the relation between *eve* stripes and gap genes in the posterior is rather different: for instance, *Anopheles* *eve* 6/7 are symmetrical on either side of posterior *hb* (Fig. 6 in [10]), while they are both anterior to *Drosophila* *hb*. Finally, *Anopheles* even has a weak (and late) extra 8th *eve* stripe compared to *Drosophila*.

**Fig 2:**
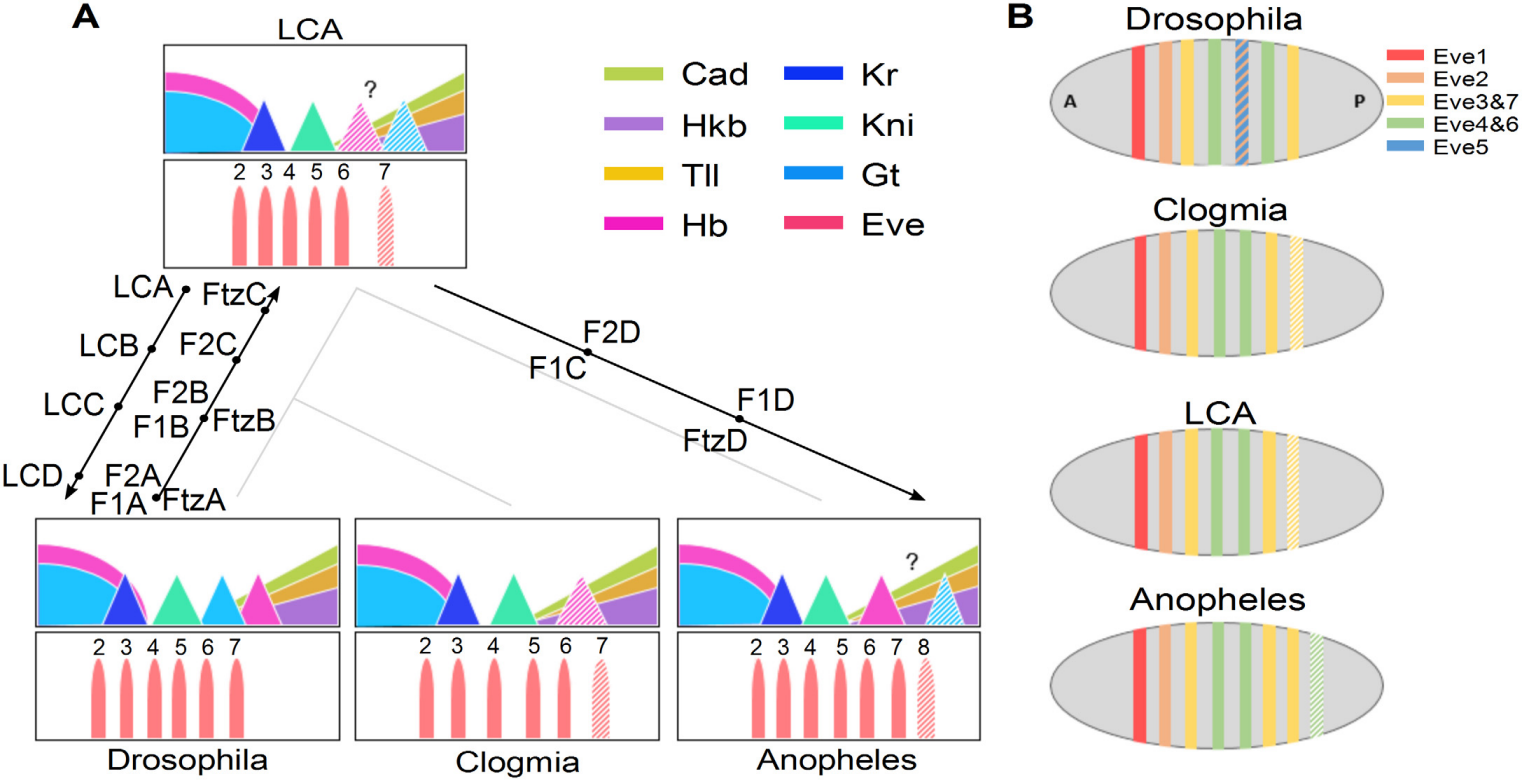
Summary of our predictions. A Predicted evolutionary pathways from different simulations detailed in Fig 3 (label F1), Fig 4 (label F2), Fig 5 (label Ftz), Fig 7 (label LC). The times shown for the intermediates are only schematic. Gap and *eve* patterns in three insect species and the inferred last common ancestor (LCA) are indicated. B Summary of homology between Eve modules in different species predicted by our evolutionary simulations.

In the following we use computation to evolve evolutionary pathways between *Drosophila* and *Anopheles* and infer a LCA. (As with simple models of molecular evolution, our mutation rates are the same forward or backward in time.) All solutions described here were found several times and for varying parameters of the viability functions or the initial network itself. S1 and S2 Videos summarize the evolutionary pathways. Predicted evolutionary pathways are displayed on a simplified insect evolutionary tree in Fig 2A.

### Variation in *eve* stripes and subsequent loss of *eve* 5

Simulations begin from the *Drosophila* network in Fig 1 and target the *Anopheles* gap pattern as an end point. The number of *eve* stripes (including 1) must be at least 7, and there is no restriction on their relative size or position.

**Fig 3:**
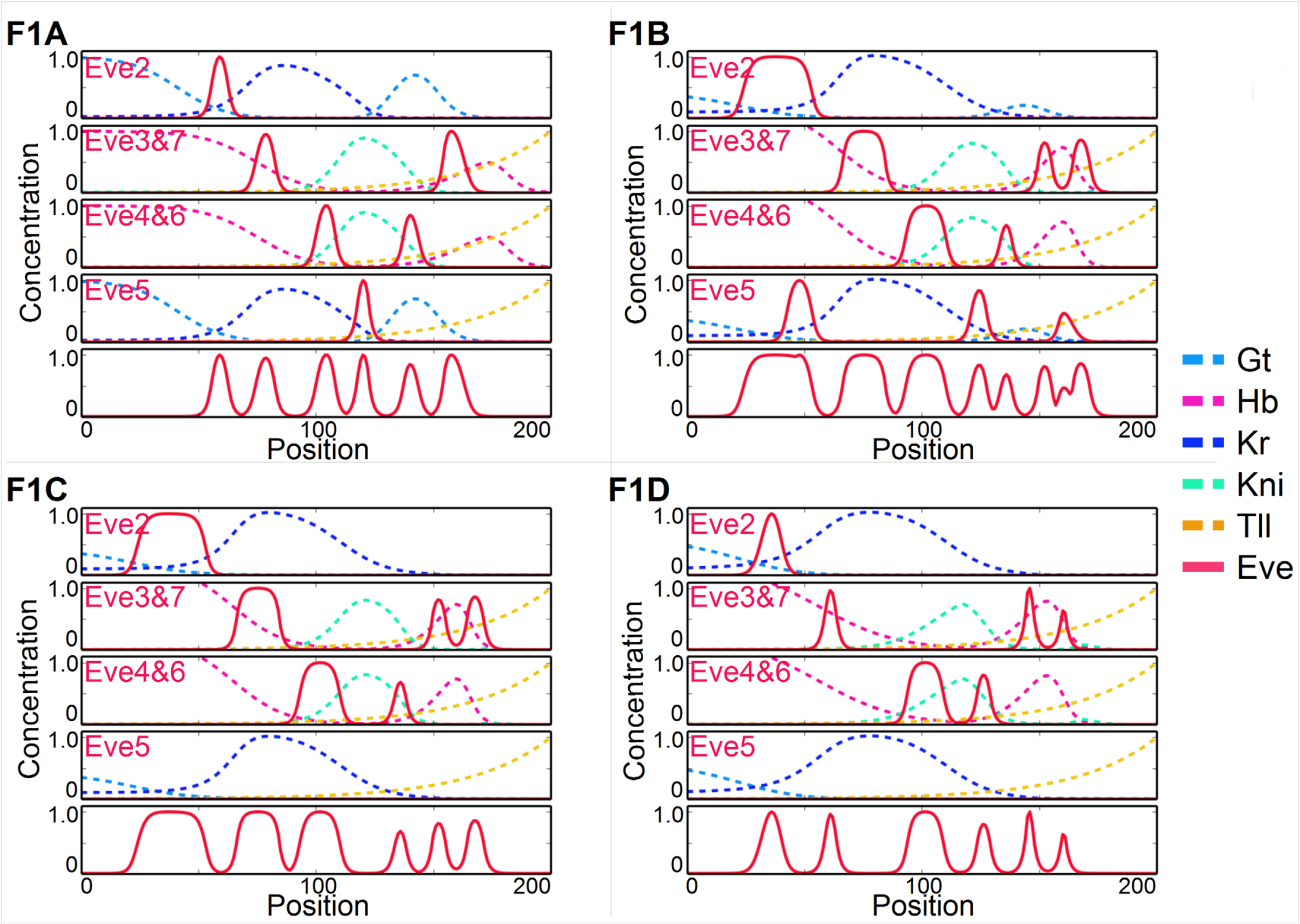
Simulated evolutionary pathway (label F1 on Fig 2A) from *Drosophila* to *Anopheles*, with salient changes discussed in the main text. For each transcriptional *eve* module only the gap genes that regulate it are shown with the same color scheme as Figs 1 and 2A. *eve* stripe 1 is not shown and the maximum expression of each module is normalized to 1 except when it dips beneath a threshold equivalent to its loss.

A typical example of such simulation is provided on Fig 3, with intermediate steps pictured on the phylogeny in Fig 2A. As the posterior *hb* domain moves forward it splits *eve* stripe 7. The repression from *hb* that defined the posterior boundaries of *eve* 6–7 gradually shifts to *tll*. Stripe 5 transiently fragments into two additional domains, Fig 3B, neither of which emerges as an distinct stripe. But once *eve* 7 splits in two, the stripe 5 element can disappear while respecting our constraint of at least 7 stripes. After it disappears posterior *gt*, is superfluous.

**Fig 4:**
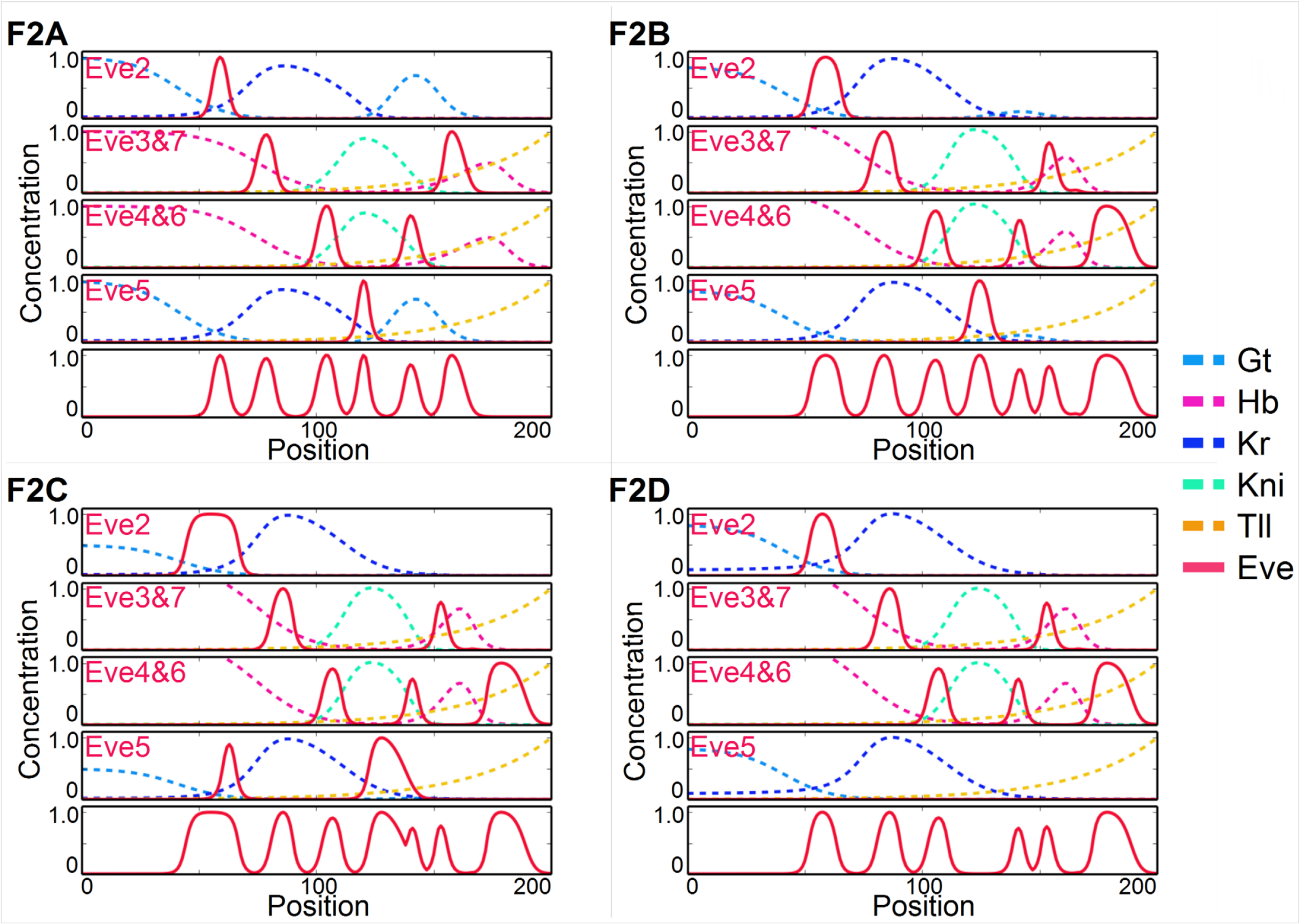
Simulated evolutionary pathway (label F2 on Fig 2A) from *Drosophila* to *Anopheles*, following the conventions of Fig 3. In C the posterior *eve* 5 stripe is counted as merged with stripe 6.

A variation on this pathway is presented on Fig 4. This time the evolution of the posterior *hb* domain anterior, splits *eve* 6 to create a new *eve* 8. Once a new *eve* stripe appears in the posterior (Fig 4B), posterior *gt* first disappears so that *eve* 5 expands posteriorly, fusing with *eve* 6 (thus effectively disappearing, (Fig 4C). Thus the *eve* 5 stripe module is no longer needed and disappears, leading to a final configuration similar to Fig 3D.

The evolutionary scenario with creation of a new *eve* stripe in the posterior and subsequent removal of *eve* 5 and *gt* posterior is highly reproducible in our simulations for a variety of conditions that implement the same evolutionary pressures. Thus stripes 4+6 and 3+7 in *Drosophila* become stripes 4+5 and 3+6 in *Anopheles*. Furthermore one of these *Drosophila* modules controls 3 stripes in *Anopheles*. On Fig 3, *Drosophila* *eve*3+7 gives rise to stripes 3,6,7, while on fig 4, *Drosophila* *eve*4+6 gives rise to stripes 4,5,7. In both cases, one *eve* stripe is split by *hb* to give two stripes in the posterior that are symmetrically positioned around the *hb* domain. The 8 stripes in *Anopheles* would be most easily explained if both modules grow another stripe with *Drosophila* *eve* 4+6 (resp. 3+7) becoming 4,5,8 (resp. 3,6,7) in mosquito.

Our simulations also predict that intermediate dipterans must have retained this logic where *eve* 4+5 (resp. 3+6) are homologous to the *eve* 4+6 (resp. 3+7) module in *Drosophila*. Both modules are repressed by *kni* and *hb*, so in particular stripes 4–5 and 3–6 should be laid symmetrically with respect to *kni* in those intermediate dipterans. This is a prediction of our computation, that exploits the known gap gene regulation, but was in no way imposed. Strikingly, *eve* stripes 4+5 and 3+6 in both *Clogmia* and *Anopheles* are indeed laid rather symmetrically with respect to *kni* contrary to the situation in *Drosophila*. Furthermore *Clogmia* has only 6 *eve* stripes prior to gastrulation and consistent with our model, lack the posterior *hb* domain that generated the two additional posterior stripes in *Anopheles*.

### Keeping segmentation logic by including *ftz*

We could not find examples of viable mutant flies missing 2 consecutive segments (corresponding to one full pair-rule period). This suggests that, even if in some mutants (e.g. the *hopscotch* mutant [32]), when one *eve* stripe disappears, the embryo needs to keep some polarity information required for the definition of parasegments.

Since *eve* 5 overlaps and defines A4p and A5a, proper parasegment definition means that the polarity of A4a and A5p must be maintained (and subsume cells that were in A4p and A5a). A natural hypothesis is then to assume another pair rule gene, out of phase with *eve*, must persist when *eve* stripe 5 disappears to provide input to the segment polarity system. We chose to add *ftz* to our model. We recognize that the proximate input to the segment polarity genes is not directly from *eve* and *ftz* but we have to insist that the model respect the minimal information logically required for the segment polarity pattern. Thus when an *eve* stripe disappears, the neighboring *ftz* stripes merge and only one parasegment disappears. (We will consider below how the constraints on evolution imposed by the other primary pair-rule genes in *Drosophila* [5] can be satisfied, if we insist that the relative phase among the pair rules genes is maintained.)

In a first round of simulations where we model *ftz* as a primary pair-rule gene (and postulate a pure *ftz* stripe 4 module delimited by *hb* on its anterior side and *gt* posteriorly), the evolutionary pathway observed in the previous section dies. There are several reasons for this: first *gt* controls both *eve* 5 and the putative *ftz* 4, so it is very difficult to have it disappear given this dual role while keeping the *ftz/eve* alternation that we impose. Second, if a new *eve* stripe appears in the posterior as before, it has no reason to be coupled properly to a corresponding alternating *ftz* stripe. Essentially, if *ftz* is primary, simulations fail to evolve new *eve* posterior stripes without breaking the alternation of *ftz*/*eve*.

**Fig 5:**
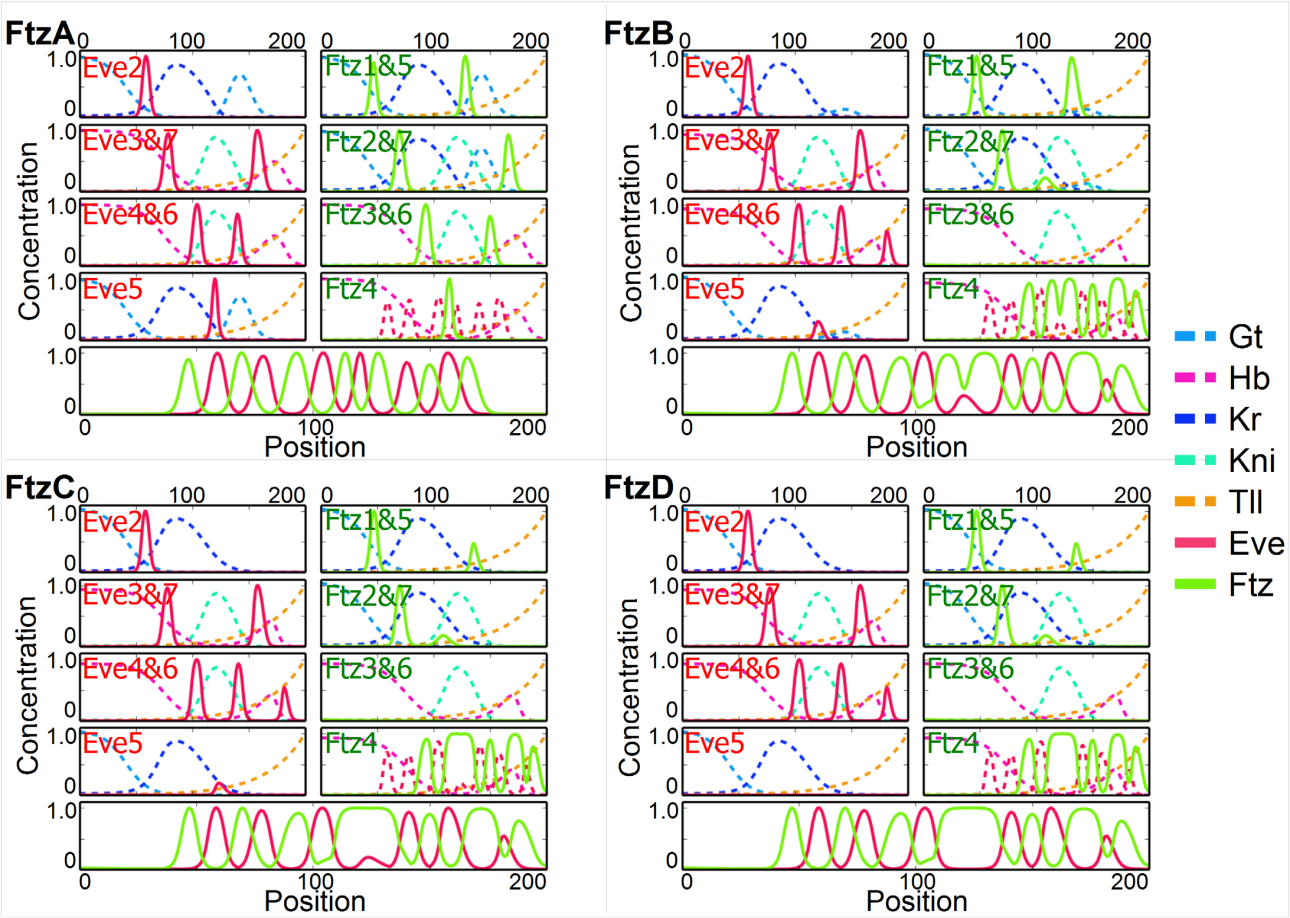
Simulated evolutionary pathway (label *ftz* on Fig 2A) from *Drosophila* to *Anopheles*, including *ftz*. Conventions of Fig 3 for *eve* and *ftz* stripes are used.

It is thus interesting in this context that *ftz* stipe 4 appears only together with the 7 stripe zebra element [5]. Thus if we allow repression of *ftz* 4-zebra by *eve* and not *gt*, it becomes slaved to *eve* in the posterior and functions as a secondary pair rule gene. In the simulation, Fig 5, *ftz* stripes 5,6,7 that are positioned by gap genes, gradually disappear in favor of the zebra element. The modules that controlled pairs of stripes 1+5, 2+7 and 3+6 now control only the anterior member and can evolve to interdigitate with the *eve* stripes. With the posterior *ftz* stripes controlled by *eve* repression, the pattern can evolve to the *Anopheles* configuration as before while preserving *eve* and *ftz* alternation throughout, Fig 5. When *eve* 5 disappears *ftz* 4 and *ftz* 5 merge (since *eve* repression is keeping them distinct) Fig 5B and 5C, thus preserving the *eve*, *ftz* alternation.

### Is the ancestral pair-rule patterning dynamically generated?

The fact that our evolutionary simulations fail when *ftz* is purely primary and succeed when *ftz* is more secondary suggests that it will be the same for other primary pair-rule genes such as *h* and *runt*. We nevertheless need to ask how the relative phase of the primary pair rule could be conserved in the evolutionary scenarios presented here. We propose, by means of a quantitative model, that pair-rule regulation in the posterior of the LCA is more dynamic than conventionally assumed in *Drosophila*. This will imply that there is no homology between the posterior regulation of the pair-rule genes by gap genes, other than for *eve* itself.

Specifically both in *Drosophila* [40] and *Clogmia* [24]*eve* stripes move from posterior to anterior prior to gastrulation. Our idea is that suitable combinations of strong and weak repression among *run*, *h*, *ftz*, and *eve* can read this phase information and stabilize the pair-rule pattern we observe in *Drosophila*, without direct gap gene input. The model is related to the pair-rule gene oscillator that patterns the posterior of short germ insects, as previously suggested in [41]. (However the model is not capable of intrinsic oscillations since *eve* is driven by gap genes and not by other pair rule genes, though it is easy to envisage how intrinsic pair rule feedback on *eve* could be gradually replaced by extrinsic gap regulation during the short to long germ band transition.) Viewed within a single cell, the forward displacement of *eve* appears as one complete temporal cycle, thus a gene regulatory network derived from a delayed negative feed back oscillator among the pair rule genes can use the same interactions to produce stable phases in space. In certain respects our conjectured LCA resembles *Nasonia* [42] where the segments posterior to A5 are patterned dynamically as we reconsider in more detail in the Discussion.

**Fig 6:**
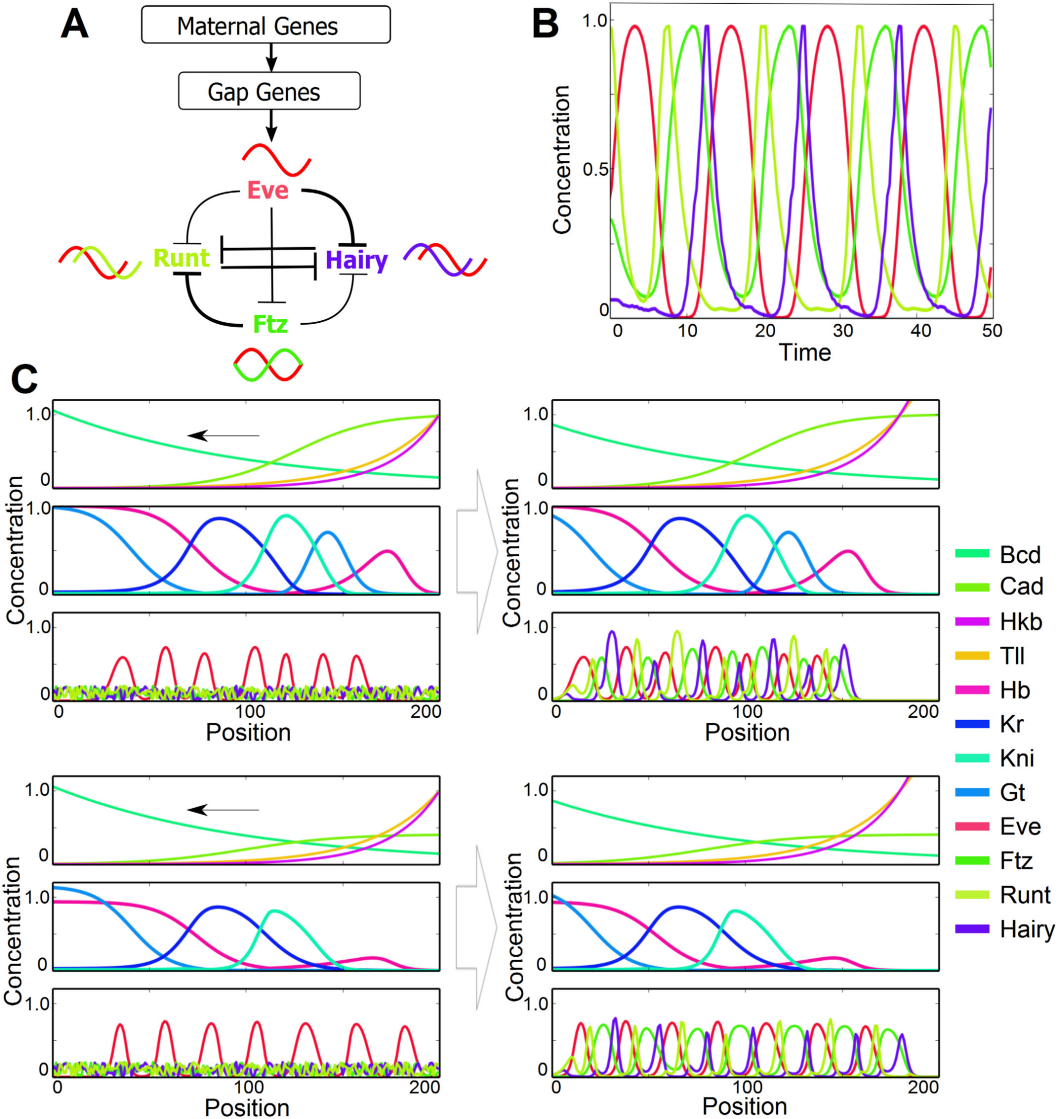
A model for the LCA that imparts stable phase relations among the pair-rule genes and remains consistent with the evolutionary pathway from fly to mosquito. (A) Schematic of the model showing gap input to eve only. The intensity of repression among the remaining genes (chosen arbitrarily as the primary pair-rule genes in fly) is shown by the line intensity and defines their relative phase. (B) Behavior of the model in A in response to imposed temporal oscillations of Eve, showing phase relationships between different pair-rule genes. Viewed within a cell, one cycle of temporal oscillations would result from the forward shift of the entire Eve pattern by one period. (C,D) If we implement a forward shift of *eve* by one stripe (left to right panels), by suitably scaling the maternal gradients, then an arbitrary initial arrangement of the three remaining genes is reset to the proper phasing for fly. We show the gap gene configuration for fly in (C) and for mosquito in (D). S3 and S4 Videos show the evolution from the left to right panels respectively for panels (C) and (D). For simplicity only, the model is applied across the entire embryo, though in reality the anterior gap gene input to the primary pair rule genes can remain invariant.

To implement our model, we control the maternal gradients to move the gap-genes forward and they drag *eve* along with them. The maternal gradients are adjusted to induce a forward shift of precisely one period in the *eve* pattern, Fig 6. Then for both the *Drosophila* and *Anopheles* gap gene patterns, the interactions shown in Fig 6A will direct an arbitrary expression pattern for the pair-rule genes other than *eve* to stably assume the relative phases we know for *Drosophila*, (or any other one by adjusting the strengths of repressions, see S3 and S4 Videos). For demonstration purposes only, we applied our model to the entire anterior-posterior axis, though we expect as in Fig 5 that the anterior gap gene regulation can persist.

More detailed models of the gap gene network reproduce directly the anterior shift that we put in by hand [9, 35]. However they take as input the dynamic maternal gradients (in particular *cad*), and further observe that *bcd* itself is dynamical, which is consistent with what we assumed. A difference is that their models, along with [23], aim to reproduce precise developmental dynamics, while we have sacrificed this level of detail and prefer to reveal parsimonious phenotypic mechanisms that have a greater claim to validity over the large evolutionary distances we cover.

### From LCA to *Drosophila*: Creation of *eve* 5

In our simulated evolution from *Drosophila* to *Anopheles*, *eve* 5 always disappeared. Thus an important consistency check is to show how *eve* 5 can appear when evolving from a LCA as appears in Figs 3 and 5C, to *Drosophila*. The solution was already suggested in Fig 4C, when a weak *eve* 2 stripe emerged from the stripe 5 module. Indeed these two stripes share the *Kr* and *gt* repressors.

**Fig 7:**
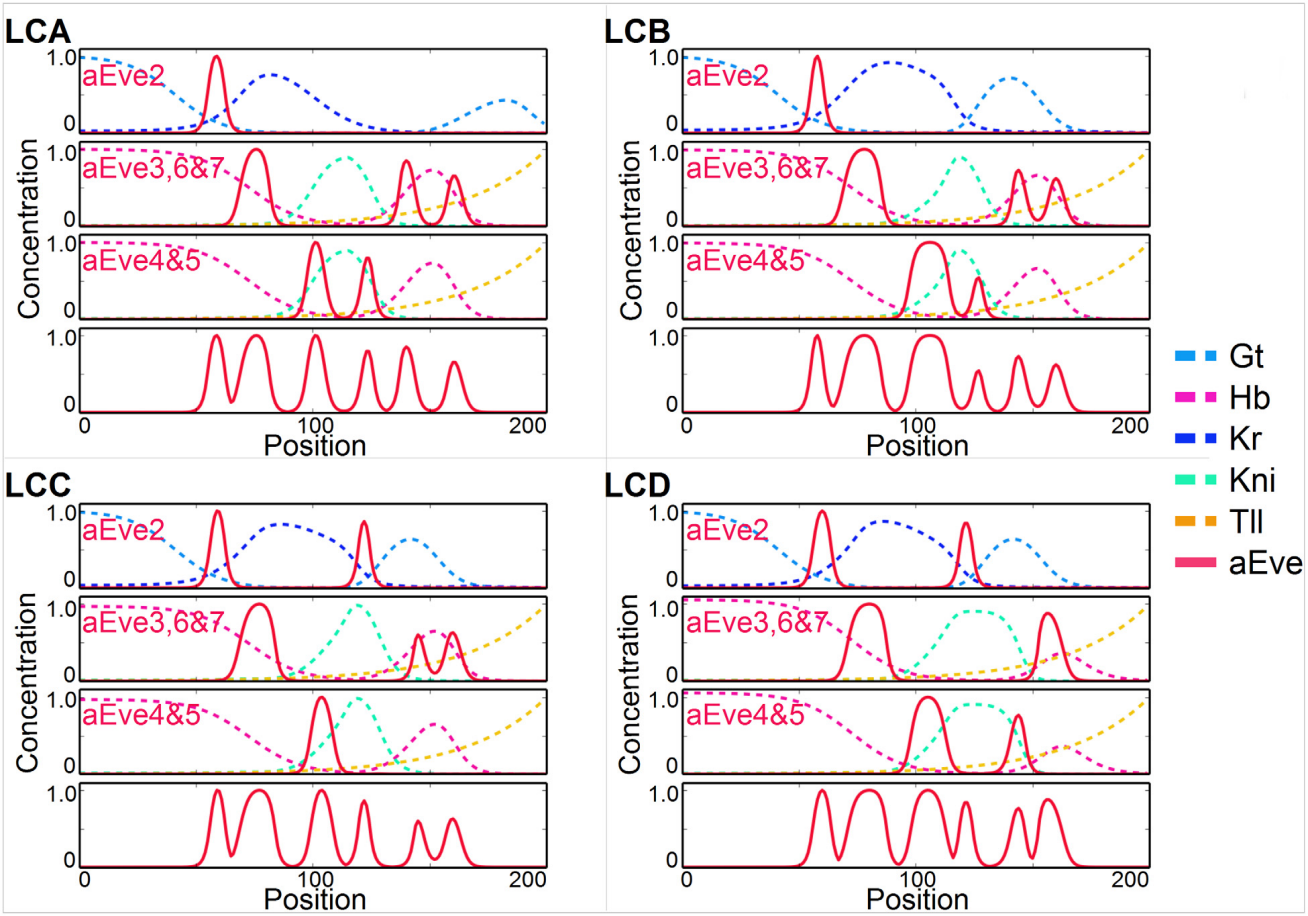
Simulated evolutionary pathway (label LC on Fig 4) from a presumptive LCA back to *Drosophila*. 4 steps of evolution are shown (A-D), details are given in the main text. Gap genes profiles are shown as well as activity of different transcriptional *eve* modules. *eve*1 is not simulated.

In Fig 7, we indeed see that when the posterior *gt* domain moves anterior the *LCA-eve* 2 module develops a second stripe, (and we imagine a distinct stripe 5 later evolves by drift). The tripartite 3,6,7 stripe loses its last component as *hb* moves posterior, and a reasonable *Drosophila* pattern is restored. Our LCA will generate a *Clogmia* like pattern if we remove posterior *hb*, illustrated on ??. There also is one *eve* stripe less than our presumptive LCA and *Drosophila*, because *hb* is not here to split ancestral *eve* 3–6 module in two in the posterior. Both these features qualitatively correspond to the observed pre-gastrulation *Clogmia* pattern, with only 6 stripes (vs 7 in *Drosophila*).

## Discussion

We have used computational evolution and the observed gap gene patterns in *Drosophila* and *Anopheles* to suggest how the gap and pair-rule network evolved between these species and their LCA as well as the relation to *Clogmia*. Our strongest conclusions are that *Anopheles* *eve* stripes 3,6,7 (resp. stripes 4,5,8) are derived *Drosophila* *eve* modules 3+7 (resp. 4+6) by the elimination of *Drosophila* *eve* stripe 5, and the forward shift and renumbering of stripes 6,7, Fig 2B. Stripes 7 and 8 in *Anopheles* are the reflection of stripes 6 and 5 respectively in the repositioned *hb* domain. The *Clogmia* pattern follows *Anopheles*, except for the elimination of stripes 7,8 which are generated by the posterior *hb* domain, which is absent in *Clogmia*. *Drosophila* *eve* 5 arises from stripe 2 of the LCA.

Our model is formulated entirely within a phenotypic or genetic description of the regulatory network, yet generated surprising, but after the fact, plausible predictions. The homologies between the *eve* stripe 3+6 and 4+5 modules in *Anopheles* or *Clogmia* and *Drosophila* could have been guessed from their symmetries around the *kni* domain, but we are not aware of a reference to that effect. But it could not be guessed that the continuous transition from *Drosophila* to *Anopheles* could be accomplished merely by adjusting the parameters within the regulatory kernels defined by *Drosophila*. This is surely the most parsimonious route between fly and mosquito, and perhaps the most rapidly evolved since it only requires mutating binding sites in existing gene regulatory modules which we know to be rapid [19, 43]. The complex structure of *Drosophila* regulation, such as duplicate enhancers, only becomes implicated in the evolutionary transition if they were found to persist in intermediate species. We can never preclude more complex scenarios, such as new modules, but their rate of evolution is uncertain in the absence of positive selection. Thus our computation is a useful heuristic tool to show that the desired transition can be accomplished by reparametrizing existing kernels without creating new ones, via evolutionary *bricolage* [20].

The most immediate tests of our predictions require identifying pair-rule gene regulatory modules in *Anopheles* and in fact none has been found in that species or *Clogmia* to our knowledge. The most expeditious route to their discovery, with modern technology (e.g. [44]) would be CHIP-seq with antibodies against the gap genes. Putative binding sites could be refined computationally and then clusters of them could be matched numerically against the regulatory regions of relevant genes, [45, 46]. In *Drosophila*, computationally defined clusters of binding sites were very successful in reconstructing gap and pair-rule regulation and this approach could in principle be applied to other species. Our first prediction is the existence of *eve* modules in *Clogmia* with purely *kni* *hb* and *tll* binding sites and suggestive of their expression as stripes 3+6 and 4+5, and the analogous prediction of putative 3-stripe modules in *Anopheles*. A more dramatic confirmation of theory would be observing the expression of these modules in *Drosophila*. This requires sufficient homology between the gap gene proteins, which is not a given, since computational screens of these other genomes with the *Drosophila* binding site weight matrices has yielded nothing. However the conservation of enhancer function between *Drosophila* and *Tribolium*, in several cases gives one hope [47, 48].

More speculatively, our model requires a LCA where the posterior pair-rule genes other than *eve* derive their phasing from the anterior shift of *eve* observed in *Drosophila* and *Clogmia* [24, 40]. The connection between an anterior shift of the posterior pair-rule genes and a segmentation clock was made in the discussion of a paper that revealed that mechanism in *Tribolium*, [41], but is speculative. Our model concerns only long-germ dipterians.

Recent work on *Nasonia*, a long-germ band *Hymenoptera*, provides a very informative bridge between short and long germ band insects and the state we impute to our LCA. *Hymenoptera* is an out-group for the order *Diptera* considered here [42, 49]. *Nasonia* gap/*eve* pattern is qualitatively very similar to *Drosophila* in the anterior part of the embryo [50, 51], precisely until abdominal segment A5 (corresponding to *ftz* 5 in fly) [42] which is the position where some strong variability between species in gap/*eve* is observed in our simulations. Posterior to this segment, *Nasonia* pair-rule pattern presents all the characteristic of an insect segmentation clock, with *eve* on the top of the hierarchy controlling waves of expression of *odd* [42]. (Note *eve* has the segmental period in the posterior, as also seen in the centipede *Strigamia* [52].) Other pair-rule genes necessary to set proper segment polarity appear downstream of this clock system [42].

We chose *ftz* as the second pair-rule gene in the simulations to define the 14 parasegments since it regulates *engrailed* and has its zebra regulatory element that allowed the simulation to position the posterior *ftz* stripes by repression from *eve*. *odd* might seem a more logical choice to define the 14 parasegments, since it is part of the posterior oscillator in short germ insects, but the simulation would encounter the same difficulty as found for *ftz*, namely it is impossible to find paths for posterior *hb* and *gt* that are compatible with the known gap gene regulatory kernels in *Drosophila* and preserve the pair-rule gene alternation.

If we combine the phylogenic evidence from *Nasonia* with our inability to evolve multiple pair-rule genes with purely gap gene regulation, then an alternate conceptual distinction between primary and secondary pair-rule genes naturally arises, along the lines already suggested in [53] using data from *Strigamia* (see also data from *Glomeris* [54]). Primary pair-rule genes are those involved in the posterior segmentation clock, the secondary genes take input from the primary and control the segment polarity layer. Delayed negative feedback is a natural way to build an oscillator with a stable period. If the segmentation clock operates by phased sequential repression among the primary pair-rule genes then the same repression could operate in space, anterior to the oscillating growth zone, to fix the relative position of these same genes with the same relative phases. (A related conversion of a temporal signal to a static one was derived in a prior study on the evolution of Hox patterning during the short to long germ transition [55].) This is a prediction that could be tested in *Tribolium* [56]. We have assumed that in the ancestral short to long germ transition (or fly to mosquito), it is *eve* that first acquires gap gene input and breaks the negative feedback oscillator, based on circumstantial evidence, but this is not a logical necessity of the model.

If we are correct that the LCA used the anterior shift of *eve* to set the relative phase of the other pair-rule genes, then the posterior regulation of these genes by the gap genes would be recent and derived, and it should not be the basis for classifying primary vs secondary. Thus we would not expect any homology between the posterior gap gene input to the pair-rule genes other than *eve* in fly and mosquito. This proposal is difficult to test since convergent evolution is a real possibility here, since any module will use the gap genes that are appropriately positioned for its regulation.

Most *evo-devo* studies involve close enough species that there is no question that intermediates are viable. However the LCA of fly and mosquito was more than 200 million years ago [57] and we are proposing an evolutionary chain of events in the blastula and presuming viable adults exist along the way! The best evidence we can offer, is the *hopscotch* mutants [32] (a component of the Jak-Stat pathway). A maternal hypomorph rescued by a wildtype male, loses A5, yet gives rise to fertile flies of both sexes. So in this mutant, no essential part of the anatomy is lost with A5. The gap gene expression is unaffected, but stripe *run* 5 is absent, and *eve* 3/5 are suppressed, *eve* 5 more so than *eve* 3 [33]. The embryo tolerates other abdominal segment loss, e.g., reduced expression of *eve* 4/6 results in loss of two abdominal segments but viable adults [31]. If we consider the Hox genes as the basic mediators of segment identity then based on expression, abdominal segments 2–7 are identical [58], but more subtle differences in Hox regulation remain [59]

Our modeling differs from earlier work that focused more on the developmental dynamics of gap gene expression in *Drosophila*, such as [8, 9]. As noted in [9], fitting dynamic data will be difficult to scale up for more complex pathways, and these authors did not consider the pair rule genes. Thus it might prove challenging to study significant evolutionary changes with such detailed models. Our coarse-grained description, relying on minimal interaction (in a spirit similar to [23]) allows us to model long evolutionary time-scales, moving from microevolution to mesoevolution [60].

Our approach illustrates the interest of phenotypic models for evolutionary systems biology. It gives a quantitative framework to make qualitative predictions (such as “one stripe appears in this region while one stripe disappears in another region”) using semi-quantitative phenotypic data directly obtained from experiment (here, gap gene positioning and constraints on stripe number/alternation). The ability to generate novel predictions directly from currently available measurements is thus of interest for a broad swath of biological modeling [21, 27].

## Supporting Information

S1 Video From *Drosophila* to *Anopheles*, a film of the best networks determined by the fitness along the evolutionary pathway from *Drosophila* to *Anopheles*, corresponding to Fig 5 in the main text. (AVI)

S2 Video From the last common ancestor to *Drosophila*, a film of the best networks determined by the fitness along the evolutionary pathway from the last common ancestor to *Drosophila* corresponding to Fig 7 in the main text. (AVI)

S3 Video *Drosophila* segmentation gene shift, adding *hairy* and *runt* to the network of *Drosophila*, a shift of the maternal gap genes towards the anterior is introduced then stopped. The gap and pair-rule genes downstream shift forward by one *eve* stripe and in the process generate the known phasing among the primary pair-rule genes.
(AVI)

S4 Video *Anopheles* segmentation gene shift. We took as initial pattern a network similar to our evolved *Anopheles* network and adjusted parameters to have stripes or equal size. Then, similar to *Drosophila*, after adding *hairy* and *runt* to the network of *Anopheles* a shift of the maternal gap genes towards the anterior is introduced then stopped. The pair-rule genes again acquire a defined phasing, starting from arbitrary initial conditions.
(AVI)

S1 Text S1 Text describes mathematical formalism and assumptions for *Drosophila* initial network, describes the fitness used, and details numerical implementations of kernels/networks as well as associated C codes. (PDF)

S1 Fig S1 Fig illustrates the presumptive LCA network (A) without and (B) with *hb* posterior domain. Interestingly, *kni* and downstream genes in the cascade are also affected by this change. In the last common ancestor profile with *hb*, Fig 5B, the ancestral *eve* 6 and 7 are found symmetrically positioned on each side of the *hb* posterior peak. In Fig 5A, the deletion of this concentration of *hb* combines these stripes and the subsequent extension of *kni* condenses the form into a single *eve* stripe, obtaining a profile qualitatively similar to *Clogmia*.
(PNG)

S1 Codes This zip folder contains C codes with parameters of the networks described in the main tex. See S1 Text for more details. (ZIP)
