# Supplementary material for: Predicting Ancestral Segmentation Phenotypes from Drosophila to Anopheles Using In Silico Evolution
Source: PLoS Genet. 2016 May 26;12(5):e1006052. doi: 10.1371/journal.pgen.1006052 (PMC4882032; doi:10.1371/journal.pgen.1006052)
Supplement: S1 Text — (PDF) [file pgen.1006052.s005.pdf]

Supplementary Text

April 14, 2016

1 Network modelling

1.1 Mathematical formalism

We use simplified phenotypic models of transcriptional networks similar to [1] . Activity of transcriptional kernels is a function of both activator and repressor concentrations. Transcriptional regulations are modeled by Hill functions. We assume that an "OR" function is implemented for activators, while repressors act multiplicatively. For instance if kernel m is activated by two activators A_1 and A_2 and repressed by one repressor R, corresponding transcriptional activity is

$$t_m = \rho_m MAX\left(\frac{A_1^n}{A_1^n + A{*}_1^n}, \frac{A_2^m}{A_1^m + A{*}_2^m}\right)\frac{R{*}^k}{R^k + R{*}^k} \quad (1)$$

where A_1, A_2, R are concentrations of corresponding proteins, n, m, k Hill coefficients, $A{*}_1, A{*}_2, R{*}$ Hill thresholds, and ρ_m the maximum transcriptional activity. Full differential equation for corresponding protein P with transcriptional activity t_m is

$$\frac{dP}{dt} = t_m - \delta_p P + \Delta_P \frac{\partial^2 P}{\partial x^2} \quad (2)$$

where δ_p is degradation constant associated to P and Δ_P diffusion constant. For the results described here diffusion does not matter and can safely be taken to 0.

Typically, gap genes positioning can be well captured with one kernel t_m per gap gene. For pair-rule genes (*eve*, *ftz*) we need to have several independent stripe kernels. These kernels were modelled as independent 'genes', and then combined with a max function to represent the corresponding pair-rule gene. In particular, this allows us to study evolution of individual kernels and thus infer kernel homologies.

Considering *eve* as an example, each stripe kernel is described by Equations 1-2 with the appropriate activators and inhibitors and zero diffusion constant. The output of each module is then fed through an additional Michaelis-Menten expression to normalize the various kernels to a common scale and the result combined with the same "OR" function as before.

$$t_{eve} = MAX(\frac{[eve2]^{n_1}}{[eve2]^{n_1} + C_1^{n_1}}, \frac{[eve5]^{n_2}}{[eve5]^{n_2} + C_2^{n_2}}, \frac{[eve3\&7]^{n_3}}{[eve3\&7]^{n_3} + C_3^{n_3}}, \frac{[eve4\&6]^{n_4}}{[eve4\&6]^{n_4} + C_4^{n_4}}) \quad (3)$$

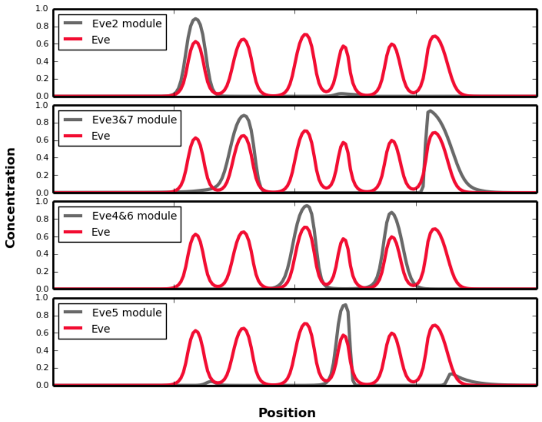

Figure ST1: The individual modules of *eve*, depicted in grey, activate the final common *eve* pattern, in red.

$$\frac{d[eve]}{dt} = t_{eve} + \Delta_{[eve]} \frac{\partial^2 eve}{\partial x^2} - \delta[eve] \tag{4}$$

and similarly for ftz. This is illustrated on Figure ST 1 for *eve*. Initial parameters were adjusted by hand to give relative gap genes/stripe positioning corresponding to the observed *Drosophila* phenotype.

1.2 Idealized *Drosophila* network

The starting point of all our simulations is an idealized version of the *Drosophila* network.

Maternal genes are the Input of our system, and define two gradients : an anterior one, *bcd* in *Drosophila*, and a posterior one, *cad*, repressed by *bcd* . *bcd* does not exist outside of *Drosophila*, so for evolutionary simulations, we will assume that this anterior maternal gradient corresponds to another maternal gene (such as *otd*, which has been simply replaced by *bcd* in the evolutionary pathway leading to *Drosophila* [2]). To fully define downstream positional information, we need to account for two other posterior gradients *tailless* and *huckebein* (*tll* and *hkb*, see below).

For gap genes, we make the following minimal assumptions, consistent with experimental data :

- *hb* anterior domain is activated by *bcd*. *hb* posterior domain regulations are still not fully resolved. This is a case where we need to assume that some positional information is transmitted by posterior gap genes, and we will thus assume *hb* posterior domain is activated by a generic posterior gap genes (we chose *tll* as suggested in [3]), and repressed by another posterior gap gene such as *hkb* [4].

- *gt* is assumed to be activated by *bcd* anteriorly and by *cad* posteriorly [5], and repressed by *Kr*.

- *kni* is activated by *bcd* and repressed by *hb* [5] [6]
- *Kr* is activated by *bcd* and repressed by both *gt* [7,8] and *hb* [9]

This chosen set qualitatively and minimally recapitulates the anterior-posterior order of expression of gap genes in our simplified model. While there are other known mutual repression betweeen gap genes (e.g. *kni* is known to repress *hb*), they are not crucial to define the relative positioning of the domains and for simplicity we have not included them.

Regulation of pair-rule genes are described in the main text. The parameters were chosen to establish an initial profile that was similar to the qualitative profile in the *Drosophila* embryo.

We simulate our embryo as a linear array of 200 cells. The profiles of *bcd*, *tll*, and *hkb* are fixed to match *Drosophila* and do not evolve in time. All other genes are initialized to zero and are integrated until they reach a steady profile. Evolution only acts on interactions downstream of *bcd*, *tll*, and *hkb*.

For simulations with ”sliding” stripes, we slowly slide those inputs numerically with time towards the anterior for a total drift of 20 cells. See Section 3 below for descriptions of actual parameters and code.

2 Network evolution

Evolution algorithm used is identical to the one used in previous works, reviewed in [10,11] (code is available upon request). It proceeds with a typical population of 50 networks. Differential equations are integrated, then networks are ranked based on their fitness (see below), and only half of the population is retained, duplicated and mutated for the next generation. For mutations, we only allow parameter variations. In particular, network topology is conserved.

Selective pressure is encoded in a fitness function that is minimized, by analogy with energy in physics. Below we describe the fitnesses used.

2.1 Fitness 1: from *Drosophila* to *Anopheles*

To find an evolutionary pathway between *Drosophila* and *Anopheles*, we require that

- network has to maintain to at least 7 stripes, since this is consistent with both the initial and final profiles.
- if ftz is simulated, the model must alternate expression of the segmentation genes eve and ftz.

Thus if either of those conditions are not met, the network is irrelevant and assigned a high (i.e., poor) score.

The embryo is modeled as a linear array of cells indexed by an integer i, $i = 0$ being the anterior, $i = N = 200$ the posterior. Expressing the concentrations of $gt(i)$ in cell i , we compute $diff_{gt} = \sum_{i>N/2} gt(i)^2$. Since there is no *giant* in the posterior of the mosquito [12], we want to minimize this sum.

Similarly for hb we compute the difference between the current profile $hb(i)$ and a predefined profile (qualitatively similar to *Anopheles*) $hb_{ano}(i)$ $diff_{hb} = \sum_{i>N/2}(hb(i) - hb_{ano}(i))^2$.

Finally, the difference of the current *eve* profile and the initial *eve* profile in the anterior can be added to the fitness with a similar term $diff_{eve}$, to keep a regular *eve* profile in the anterior ($i < N/2$, which corresponds to *eve* 2-4 stripes with our choice of coordinates).

The fitness score (to be minimized) used for simulations in the main text of the paper is:

$$Score = C_1 diff_{gt} + max(C_2 * diff_{hb}, C_3 * diff_{eve}) + \begin{cases} 0 \text{ if } ftz/eve \text{ alternation and } \geq 7\text{stripes} \\ 1000 \text{ otherwise} \end{cases} \quad (5)$$

The different constants C_1, C_2 and C_3 are there to scale the strength of each individual component of the score. Different values of the constants were tested across many simulations but do no yield strong differences as long as $C_1 \sim C_2$. For the simulations shown in the main text, the values actually used were of $C_1 = C_2$=1, C_3=0.01 without ftz (C_3 can actually be taken to 0 without changing results qualitatively for those cases) and $C_3 = 1$ with ftz. Note that when simulations were conducted without ftz, the condition of alternating the stripes was removed and the rest of the fitness was maintained.

With the values of C_1, C_2, C_3 given above, around 10% of the simulations gave "successful" results, i.e. gave an evolutionary pathway with more than 7 stripes, *hb* more anterior than in initial network and no *gt* in the posterior. The remainder failed to converge in the allotted time. The important point is that our simulations always succeed in the same way as presented in the main text, via duplication of a posterior *eve* stripe and elimination of *eve*5.

2.2 Fitness 2: Last common ancestor to *D*rosophila

In that case, starting with presumptive ancestor, we define idealized fly profiles for each of the gap genes (respectively $kni_{fly}(i)$, $Kr_{fly}(i)$, $gt_{fly}(i)$ and $hb_{fly}(i)$ and for each gap gene define a mean square deviation score similar to what is done above, e.g. for hb $diff_{hb} = \sum_i(hb(i) - hb_{fly}(i))^2$. We sum contributions of all gap genes to define $diff_{gap}$. Similar to the previous case, a high score was attributed if less than 7 stripes of *eve* were observed in the embryos profile, i.e.

$$Score = diff_{gap} + \begin{cases} 0 \text{ if } \geq 7\text{stripes} \\ 1000 \text{ otherwise} \end{cases} \quad (6)$$

3 Parameters and codes for networks

Evolutionary simulations generate an enormous amount of possible parameters and networks. To illustrate this, we include in Supplementary Materials a complete set of `C` codes corresponding to the evolved networks commented in the main text. Those codes were automatically generated by our `Python` evolutionary code, and allow for simple simulations of kernels dynamics.

Each subdirectory is named after a Figure from the main text, and contains `C` codes named with corresponding panels. For instance `Fig6B.c` contains parameters for networks of Fig. 6B, and integrates corresponding equations. We include python scripts `plot_name_of_pair_rule_gene.py` encoding a simple graphical python tool to visualize the corresponding integrated profile. To run these simulations, the code should be simply compiled and run, and python plotting tool allows visualization, e.g. :

```
gcc Fig6B.c
./a.out
python plot_eve_ftz.py
```

should display a pop-up window with profiles corresponding to Fig. 6B. Code also creates a file named "Buffer0" containing values for each gene as a function of time and position, that is used to generate the plot.

Differential equations corresponding to the kernels are encoded in the function `derivC()` in each of the `C` program. This function computes derivatives of the different variables `ds` (the correspondence between index of `ds` and actual genes is in the list called `List_Genes.txt` in each corresponding directory). As an example, function `derivC()`for the initial network we used for *Drosophila* in `Fig6A.c` is :

```
 void derivC(double s[],double history[][NSTEP][NCELLTOT],int step, double ds[],double ↩
     memories[],int ncell){
 int index;  for (index=0;index<SIZE;index++) ds[index]=0;//initialization
     double increment=0;
     double rate=0;

/*************degradation rates****************/
          rate=1.000000*s[0];
          increment=rate;
          ds[0]-=increment;
          rate=1.000000*s[1];
          increment=rate;
          ds[1]-=increment;
          rate=1.000000*s[2];
          increment=rate;
          ds[2]-=increment;
          rate=1.000000*s[3];
          increment=rate;
          ds[3]-=increment;
          rate=1.000000*s[4];
          increment=rate;
          ds[4]-=increment;
          rate=1.000000*s[5];
          increment=rate;
          ds[5]-=increment;
          rate=1.000000*s[6];
          increment=rate;
          ds[6]-=increment;
          rate=1.000000*s[7];
          increment=rate;
          ds[7]-=increment;
          rate=1.000000*s[8];
```

```
        increment=rate;
        ds[8]-=increment;
        rate=1.000000*s[9];
        increment=rate;
        ds[9]-=increment;
        rate=1.000000*s[10];
        increment=rate;
        ds[10]-=increment;
        rate=1.000000*s[11];
        increment=rate;
        ds[11]-=increment;
        rate=1.000000*s[12];
        increment=rate;
        ds[12]-=increment;
        rate=1.000000*s[13];
        increment=rate;
        ds[13]-=increment;
        rate=1.000000*s[14];
        increment=rate;
        ds[14]-=increment;
        rate=1.000000*s[15];
        increment=rate;
        ds[15]-=increment;
        rate=1.000000*s[16];
        increment=rate;
        ds[16]-=increment;
        rate=1.000000*s[17];
        increment=rate;
        ds[17]-=increment;

/**************Transcription rates*****************/
     int k,memory=-1;
     memory=step-0;
     if(memory>=0){
        rate=MAX(1.000000,0.000000)*HillR(history[0][memory][ncell],0.300000,5.000000);
        increment=rate;
        ds[1]+=increment;
    }
     memory=step-0;
     if(memory>=0){
        rate=MAX(1.000000*MAX(HillA(history[0][memory][ncell],0.500000,9.000000),HillA(↩
            history[6][memory][ncell],0.400000,3.000000)),0.000000)*HillR(history[7][↩
            memory][ncell],0.450000,6.000000);
        increment=rate;
        ds[2]+=increment;
    }
     memory=step-0;
     if(memory>=0){
        rate=MAX(1.000000*MAX(HillA(history[0][memory][ncell],0.700000,10.000000),HillA(↩
            history[1][memory][ncell],0.600000,10.000000)),0.000000)*HillR(history[4][↩
            memory][ncell],0.900000,3.000000)*HillR(history[6][memory][ncell↩
            ],0.230000,7.000000);
        increment=rate;
        ds[3]+=increment;
    }
     memory=step-0;
     if(memory>=0){
        rate=MAX(1.000000*HillA(history[0][memory][ncell],0.350000,10.000000),0.000000)*↩
            HillR(history[2][memory][ncell],0.800000,4.000000)*HillR(history[3][memory][↩
            ncell],0.100000,1.000000);
```

```
        increment=rate;
        ds[4]+=increment;
    }
    memory=step-0;
    if(memory>=0){
        rate=MAX(1.000000*HillA(history[0][memory][ncell],0.240000,10.000000),0.000000)*↩
            HillR(history[2][memory][ncell],0.100000,2.000000)*HillR(history[4][memory][↩
            ncell],0.600000,4.000000)*HillR(history[6][memory][ncell],0.200000,5.000000);
        increment=rate;
        ds[5]+=increment;
    }
    memory=step-0;
    if(memory>=0){
        rate=MAX(1.000000*HillA(history[0][memory][ncell],0.450000,10.000000),0.000000)*↩
            HillR(history[3][memory][ncell],0.200000,10.000000)*HillR(history[4][memory][↩
            ncell],0.300000,10.000000);
        increment=rate;
        ds[8]+=increment;
    }
    memory=step-0;
    if(memory>=0){
        rate=MAX(1.000000,0.000000)*HillR(history[6][memory][ncell],0.350000,10.000000)*↩
            HillR(history[2][memory][ncell],0.550000,10.000000)*HillR(history[5][memory][↩
            ncell],0.018000,10.000000);
        increment=rate;
        ds[9]+=increment;
    }
    memory=step-0;
    if(memory>=0){
        rate=MAX(1.000000,0.000000)*HillR(history[6][memory][ncell],0.250000,7.000000)*↩
            HillR(history[5][memory][ncell],0.500000,10.000000)*HillR(history[2][memory][↩
            ncell],0.100000,7.000000);
        increment=rate;
        ds[10]+=increment;
    }
    memory=step-0;
    if(memory>=0){
        rate=MAX(1.000000*HillA(history[0][memory][ncell],0.080000,2.000000),0.000000)*↩
            HillR(history[4][memory][ncell],0.300000,10.000000)*HillR(history[3][memory][↩
            ncell],0.070000,10.000000)*HillR(history[6][memory][ncell],0.250000,5.000000)↩
            ;
        increment=rate;
        ds[11]+=increment;
    }
    memory=step-0;
    if(memory>=0){
        rate=MAX(1.000000*HillA(history[0][memory][ncell],0.100000,10.000000),0.000000)*↩
            HillR(history[2][memory][ncell],0.250000,7.000000)*HillR(history[6][memory][↩
            ncell],0.290000,7.000000)*HillR(history[5][memory][ncell],0.130000,5.000000);
        increment=rate;
        ds[12]+=increment;
    }
    memory=step-0;
    if(memory>=0){
        rate=MAX(1.000000*HillA(history[0][memory][ncell],0.100000,10.000000),0.000000)*↩
            HillR(history[6][memory][ncell],0.430000,10.000000)*HillR(history[3][memory][↩
            ncell],0.080000,7.000000)*HillR(history[4][memory][ncell],0.700000,7.000000)*↩
            HillR(history[5][memory][ncell],0.030000,7.000000);
        increment=rate;
        ds[13]+=increment;
```

```
    }
     memory=step -0;
     if(memory>=0){
         rate=MAX(1.000000*HillA(history[0][memory][ncell],0.100000,10.000000),0.000000)*↩
             HillR(history[6][memory][ncell],0.150000,7.000000)*HillR(history[4][memory][↩
             ncell],0.100000,10.000000)*HillR(history[3][memory][ncell↩
             ],0.460000,10.000000);
         increment=rate;
         ds[14]+=increment;
    }
     memory=step -0;
     if(memory>=0){
         rate=MAX(1.000000*MAX(MAX(MAX(HillA(history[11][memory][ncell],0.700000,5.000000)↩
             ,HillA(history[9][memory][ncell],0.700000,5.000000)),HillA(history[10][memory↩
             ][ncell],0.700000,5.000000)),HillA(history[8][memory][ncell↩
             ],0.700000,5.000000)),0.000000);
         increment=rate;
         ds[15]+=increment;
    }
     memory=step -0;
     if(memory>=0){
         rate=MAX(1.000000*HillA(history[7][memory][ncell],0.010000,10.000000),0.000000)*↩
             HillR(history[2][memory][ncell],0.040000,10.000000)*HillR(history[15][memory↩
             ][ncell],0.300000,10.000000)*HillR(history[6][memory][ncell↩
             ],0.100000,10.000000);
         increment=rate;
         ds[16]+=increment;
    }
     memory=step -0;
     if(memory>=0){
         rate=MAX(1.000000*MAX(MAX(MAX(HillA(history[12][memory][ncell],0.700000,5.000000)↩
             ,HillA(history[13][memory][ncell],0.700000,5.000000)),HillA(history[16][↩
             memory][ncell],0.700000,5.000000)),HillA(history[14][memory][ncell↩
             ],0.700000,5.000000)),0.000000);
         increment=rate;
         ds[17]+=increment;
    }

/**************Protein protein interactions*****************/

/**************Phosphorylation*****************/
 float total;
}
```

Kernel parameters for this network are summarized in Table 1 to illustrate correspondence between `C` code and parameters.

References

[1] Paul François, Vincent Hakim, and Eric D Siggia. Deriving structure from evolution: metazoan segmentation. *Molecular Systems Biology*, 3:9, December 2007.

[2] Jeremy Lynch and Claude Desplan. Evolution of Development: Beyond Bicoid. *Current Biology*, 13(14):R557–R559, July 2003.

	Regulated by								
Gene expression	*bcd*	*tll*	*hkb*	*cad*	*hb*	*gt*	*Kr*	*kni*	*eve*
cad	0.3/5	-	-	-	-	-	-	-	-
hb	0.5/9(+)	0.4/3(+)	0.45/6	-	-	-	-	-	-
gt	0.7/10(+)	0.23/7	-	0.6/10(+)	-	-	0.9/3	-	-
Kr	0.35/10(+)	-	-	-	0.8/4	0.1/1	-	-	-
kni	0.24/10(+)	0.2/5	-	-	0.1/2	-	0.6/4	-	-
*eve*2	0.45/10(+)	-	-	-	-	0.2/10	0.3/10	-	-
*eve*3&7	-	0.35/10	-	-	0.55/10	-	-	0.018/10	-
*eve*4&6	-	0.25/7	-	-	0.1/7	-	-	0.5/10	-
*eve*5	0.08/-2(+)	0.25/5	-	-	-	0.07/10	0.3/10	-	-
*ftz*1&5	0.1/10(+)	-	-	-	-	0.46/10	0.1/10	-	-
*ftz*2&6	0.1/10(+)	0.29/7	-	-	0.25/7	-	0.13/5	-	-
*ftz*4	-	0.1/10	0.01/10(+)	-	0.04/10	-	-	-	0.2/10

Table 1: Gene network parameters for *Drosophila*. Each interaction is tabulated as the $(concentration\ threshold)/(hill\ coefficient)$. Hill coefficients indicate repressions unless they are followed by a (+) which indicates an activation.

[3] J S Margolis, M L Borowsky, E Steingrímsson, C W Shim, J A Lengyel, and J W Posakony. Posterior stripe expression of hunchback is driven from two promoters by a common enhancer element. *Development (Cambridge, England)*, 121(9):3067–3077, September 1995.

[4] J Casanova. Pattern formation under the control of the terminal system in the Drosophila embryo. *Development (Cambridge, England)*, 110(2):621–628, October 1990.

[5] G Struhl, P Johnston, and P A Lawrence. Control of Drosophila body pattern by the hunchback morphogen gradient. *Cell*, 69(2):237–249, April 1992.

[6] Dorothy E Clyde, Maria S G Corado, Xuelin Wu, Adam Paré, Dmitri Papatsenko, and Stephen Small. A self-organizing system of repressor gradients establishes segmental complexity in Drosophila. *Nature*, 426(6968):849–853, December 2003.

[7] X L Wu, R Vakani, and Stephen Small. Two distinct mechanisms for differential positioning of gene expression borders involving the Drosophila gap protein giant. *Development (Cambridge, England)*, 125(19):3765–3774, October 1998.

[8] M Capovilla, E D Eldon, and V Pirrotta. The giant gene of Drosophila encodes a b-ZIP DNA-binding protein that regulates the expression of other segmentation gap genes. *Development (Cambridge, England)*, 114(1):99–112, January 1992.

[9] Dmitri Papatsenko and Michael Levine. The Drosophila gap gene network is composed of two parallel toggle switches. *PLoS ONE*, 6(7):e21145, 2011.

[10] Paul François. Evolution in silico: from network structure to bifurcation theory. *Advances in experimental medicine and biology*, 751:157–182, 2012.

[11] Paul François. Evolving phenotypic networks in silico. *Seminars in cell & developmental biology*, 35:90–97, November 2014.

[12] Yury Goltsev, William Hsiong, Gregory Lanzaro, and Michael Levine. Different combinations of gap repressors for common stripes in Anopheles and Drosophila embryos. *Developmental Biology*, 275(2):435–446, 2004.
