# Supplementary figures and images for: Predicting Ancestral Segmentation Phenotypes from Drosophila to Anopheles Using In Silico Evolution
Source: PLoS Genet. 2016 May 26;12(5):e1006052. doi: 10.1371/journal.pgen.1006052 (PMC4882032; doi:10.1371/journal.pgen.1006052)

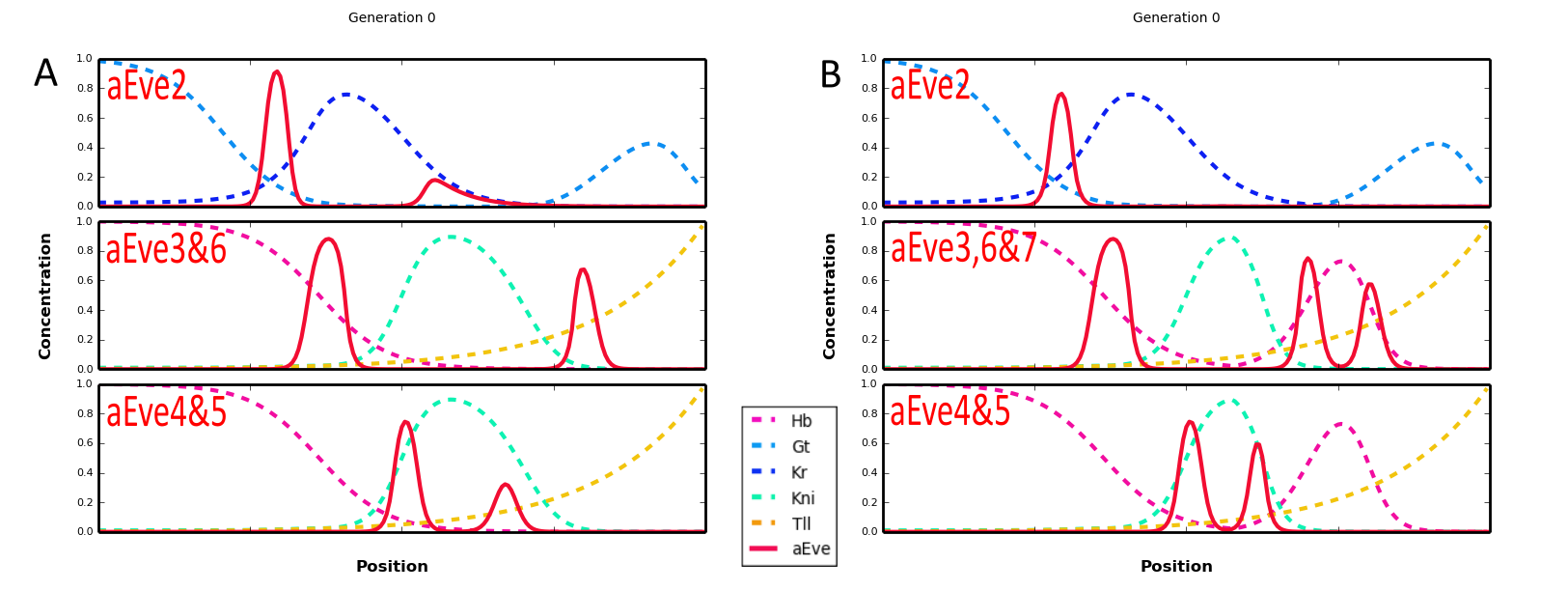

Supplement: S1 Fig — In the last common ancestor profile with hb, Fig 5B, the ancestral eve 6 and 7 are found symmetrically positioned on each side of the hb posterior peak. In Fig 5A, the deletion of this concentration of hb combines these stripes and the subsequent extension of kni condenses the form into a single eve stripe, obtaining a profile qualitatively similar to Clogmia. (PNG) [file pgen.1006052.s006.png]
